# Editorial: Bioinformatics applied to neuroscience

**DOI:** 10.3389/fnins.2023.1276346

**Published:** 2023-09-21

**Authors:** Clevia Rosset, Jaqueline Bohrer Schuch, Mariana Recamonde-Mendoza, Thayne Woycinck Kowalski

**Affiliations:** ^1^Graduate Program in Medical Sciences (PPGCM), Universidade Federal do Rio Grande do Sul (UFRGS), Porto Alegre, Brazil; ^2^Laboratory of Genomic Medicine, Experimental Research Service, Hospital de Clínicas de Porto Alegre (HCPA), Porto Alegre, Brazil; ^3^Laboratory Research Unit, Experimental Research Service, Hospital de Clínicas de Porto Alegre (HCPA), Porto Alegre, Brazil; ^4^Graduate Program in Psychiatry and Behavioral Sciences, Department of Psychiatry, Universidade Federal do Rio Grande do Sul (UFRGS), Porto Alegre, Brazil; ^5^Graduate Program in Computer Science (PPGC), Institute of Informatics, Universidade Federal do Rio Grande do Sul (UFRGS), Porto Alegre, Brazil; ^6^Bioinformatics Core, Hospital de Clínicas de Porto Alegre (HCPA), Porto Alegre, Brazil; ^7^Graduate Program in Genetics and Molecular Biology (PPGBM), Department of Genetics, Universidade Federal do Rio Grande do Sul (UFRGS), Porto Alegre, Brazil; ^8^Laboratory Genetics Unit, Medical Genetics Service, Hospital de Clínicas de Porto Alegre (HCPA), Porto Alegre, Brazil

**Keywords:** transcriptomics, systems biology, epigenetics, machine learning, neurodegenerative diseases, ischemia, autophagy, Mendelian randomization analysis

Genetics and molecular biology studies have revealed many new associations between the most diverse diseases, pointing to the involvement of a wide range of pathways. This increasing volume of data through interdisciplinary lenses and methods can provide insights into pathophysiologic mechanisms in several diseases. Therefore, new methods have arisen to analyze the data provided by these studies. Bioinformatics has emerged as a necessary discipline, revolutionizing this field and providing tools to jointly study DNA variants, gene expression, epigenetic marks, and biological networks. Biological public databases and computational tools are essential to develop data-driven approaches for understanding human diseases. This Research Topic contains 15 articles presenting current bioinformatics approaches applied to understanding the biological underpinnings of psychiatric and neurological conditions.

Dong et al. analyzed the association of immune and Parkinson's disease (PD)-related genes through protein-protein interaction networks, using co-expression data. Modular clustering analysis was also performed to identify central core genes, and the findings were validated by analyzing the expression of specific key genes. In summary, the authors were able to show strong correlations between immune- and PD-related genes, which could have the potential for diagnostic and therapeutic approaches. The integration between differential gene expression, weighted gene co-expression network analysis (WGCNA), and neurodegenerative disorders were also the topics studied by Chen et al. and He et al. The first study focused on PD, and their results also pointed to immune-related hub genes and the PD pathogenesis, which were presented as key modules in the WGCNA. He et al. focused on interconnected expression analysis and a machine learning model, through a random forest algorithm. They proposed seven hub genes that could confer the theoretical basis for studying biomarkers in Alzheimer's disease.

Other two studies used machine learning models to identify biomarkers in psychiatry and neurological conditions. Liu et al. proposed a diagnostic model of major depressive disorder (MDD) using machine learning. Data consisted of differentially expressed genes in MDD individuals and healthy controls. Most genes were involved in immune pathways and response to external stimuli. A robust diagnostic model was created through random forest and artificial neural network machine learning algorithms and potential driver genes were identified, as *C3AR1, BST2, TREM1, BTG3, LY6E*, and *IER5*. Bai et al. also used machine learning methods to identify potential biomarkers but in intracerebral hemorrhage (ICH). They examine the expression profiles of circRNAs in the peripheral blood and identify their potential functions using bioinformatic tools. Three circRNAs, named hsa_circ_0005505, circERBB2 and circCHST12, were identified as promising biomarkers for ICH based on machine learning algorithms.

Tian et al. studied for the first time the epigenetic role of m7G-regulated genes in ischemic stroke. Based on previous studies on m7G, the expression of 34 m7G key regulatory genes was searched in the datasets from the Gene Expression Omnibus (GEO), including patients and controls. Two widely used machine learning algorithms, random forest (RF) and support vector machine (SVM) were subsequently used to identify eight key regulators of m7G. Five of the eight key regulators were significantly different in the middle cerebral artery occlusion model and quantitative polymerase chain reaction validations. In summary, their findings suggest that *EIF3D, CYFIP2, NCBP2, DCPS*, and *NUDT1* genes may serve as potential diagnostic biomarkers for ischemic stroke and could predict clinical risk. Another study by Shu et al. also used the middle cerebral artery occlusion model. They identified 15 differentially expressed genes related to the three types of programmed cell death (apoptosis, pyroptosis, and necroptosis) in transcriptome signatures of brain tissue samples from mice subjected to middle cerebral artery occlusion/reperfusion (MCAO/R). They conclude that these processes and the crosstalk among them might be involved in ischemic stroke and that the key nodes and regulatory axes identified in this study might play vital roles in regulating the above processes.

Ischemic stroke and epigenetics were also the object of study of other researchers. Zhang et al. evaluated the regulators of RNA methylation in ischemic stroke and suggested therapeutic targets by applying WGCNA followed by quantitative PCR analysis in an animal model; finally, the authors performed molecular docking to predict the interaction with the hub genes and drug molecules, which identified *GPNMB* and chloroquine as potential targets. WGCNA was also applied in the study conducted by Yu et al. that evaluated chromatin regulators in ischemic stroke; the analysis pointed to four immune biomarkers (*DPF2, LMNB1, MLLT3*, and *JAK2*), which were validated through quantitative PCR and evaluated in regard to molecular docking. In a different approach, Yang et al. also evaluated immune biomarkers in ischemic stroke by studying the hub shared genes between the condition and major depressive disorder; differentially expressed genes were analyzed and it was found that innate immunity genes were upregulated whilst acquired immunity genes were downregulated.

Zhu et al. conducted a two-sample Mendelian randomization study to evaluate the association between C-Reactive Protein (CRP) levels and risk of Amyotrophic Lateral Sclerosis. Amyotrophic Lateral Sclerosis data were extracted from GWAS performed in people of European ancestry and included 20,806 cases and 59,804 controls. Six Mendelian randomization methods were selected, including the inverse variance weighted (IVW), weighted median, MR-Egger, MRPRESSO, simple mode, and weighted mode test. Fifty-seven independent SNPs were found to be associated with CRP. However, there was no significant causal relationship between genetically predicted CRP levels and disease risk (OR = 1.123, 95% CI = 0.963–1.309, *p* = 0.139), which is a relevant result since it excludes the possible association between CRP levels and Amyotrophic Lateral Sclerosis in the European population.

Autophagy was another important topic addressed. Xiao et al. investigated combinations of DGE with enrichment and systems biology analysis in intracerebral hemorrhage datasets. They suggested four autophagy-related genes, *IL1B, STAT3, NLRP3*, and *NOD2*, as key factors associated with intracerebral hemorrhage. In another study by Ma et al. autophagy was studied in the context of Alzheimer's disease through multi-omics analysis. They studied the novel ubiquitin-binding receptor, Chaperonin containing TCP1 subunit 2 (CCT2), which promotes aggrephagy, a process in which autophagy selectively degrades protein aggregates. All the datasets used in the study were obtained from the Gene Expression Omnibus database. The CCT2-high-associated genes screened by Pearson coefficients were enriched in protein folding, autophagy, and messenger RNA stability regulation pathways. The logistic prediction model screened in this study is a favorable candidate for predicting potential biological targets and small molecule inhibitors for Alzheimer's disease treatment.

Finally, two studies evaluated the role of circRNAs in neurologic conditions. The first study by Wang et al. evaluated competitive endogenous RNA regulatory networks in postoperative cognitive dysfunction (POCD). In their study, the authors extracted the transcriptomic signatures in the hippocampus of POCD mice derived from Gene Expression Omnibus (GEO) datasets in order to identify the circRNA, miRNA, and mRNA expression profiles of POCD mice compared with controls, respectively. A set of differentially expressed RNAs, including 119 circRNAs, 33 miRNAs, and 49 mRNAs were identified. Transcript validation by qPCR confirmed the enhanced expression of circ_0001634, circ_0001345, and circ_0001493. A regulatory network was constructed using circRNA-miRNA pairs and miRNA-mRNA pairs, resulting in a competing endogenous RNA regulatory network composed of three circRNAs, three miRNAs, and six mRNAs. The hub mRNAs in the network were further found to be involved in the hormone catabolic process and regulation of the canonical Wnt signaling pathway, revealing their crucial role in POCD.

The second circRNA study evaluated the potential value of differentially expressed circular RNAs derived from circulating exosomes in the pathogenesis of rat spinal cord injury (SCI). SCI remains a catastrophically injured condition for humans, thereby bringing severe social and economic burdens. The study by Zan et al. analyzed differentially expressed circRNAs derived from circulating exosomes in SCI rats in comparison with the control rats. Subsequently, functional enrichment analyses including kyoto encyclopedia of genes and genomes (KEGG) pathway and gene ontology (GO) were performed to evaluate the possible biological functions of upregulated as well as downregulated circRNAs involved in SCI. Five upregulated circulating circRNAs including and five downregulated circulating circRNAs were verified through reverse transcription-polymerase chain reaction. They also constructed a circRNA-miRNA-mRNA gene interaction network to predict the possible functionalities of circRNAs in SCI through anticipating specific interactive miRNAs. Their main findings suggest the possible involvement and functional significance of circRNAs in SCI.

The manuscripts included in this Research Topic show the diversity of bioinformatics tools that could be applied to investigate intriguing questions in the neuroscience field. They could direct more precise *in vitro* analyses and provide interesting findings to clarify the pathogenesis of complex diseases, as well as to suggest novel biomarkers and treatment options.

## Author contributions

CR: Conceptualization, Supervision, Writing—original draft, Writing—review and editing. JS: Conceptualization, Supervision, Writing—original draft, Writing—review and editing. MR-M: Supervision, Writing—review and editing. TK: Conceptualization, Supervision, Writing—original draft, Writing—review and editing.

